# Treatment response to idelalisib in a patient with immunodeficiency-associated Burkitt lymphoma harboring a *PIK3CA* H1047R mutation

**DOI:** 10.1007/s00277-020-03974-y

**Published:** 2020-03-20

**Authors:** Annalen Bleckmann, Sascha Dierks, Hans-Ulrich Schildhaus, Niels Hellige, Ulrike Bacher, Lorenz Trümper, Gerald Wulf

**Affiliations:** 1 Department of Haematology and Medical Oncology, University Medicine Göttingen, Göttingen, Germany; 2 Department of Medicine A, Hematology, Oncology and Pneumology, University Hospital Münster, Münster, Germany; 3 Department of Pathology, University Medicine Göttingen, Göttingen, Germany; 4 Department of Radiology, University Medicine Göttingen, Göttingen, Germany; 5 Department of Hematology and Central Hematology Laboratory, Inselspital, Bern University Hospital, University of Bern, Bern, Switzerland

Dear Editor,

While in Burkitt lymphoma (BL) immunochemotherapy readily induces remissions, the clinical outcomes for patients with refractory or recurrent disease are still adverse [1, 2]. The phosphatidyl-inositol-3 kinase (PI3K) signal transduction pathway is involved in the activation, proliferation, and migration of B lymphocytes, and a cooperation of MYC and PI3K has been described in BL oncogenesis. Idelalisib is a highly selective inhibitor of the PI3K delta isoform [3] with significant clinical activity against indolent B cell lymphomas [4, 5]. We here report the successful salvage therapy with idelalisib in a patient with relapsed immunodeficiency-associated BL exhibiting a *TP53* mutation and a H1047R mutation in the *PIK3CA* gene.

**Fig. 1:**
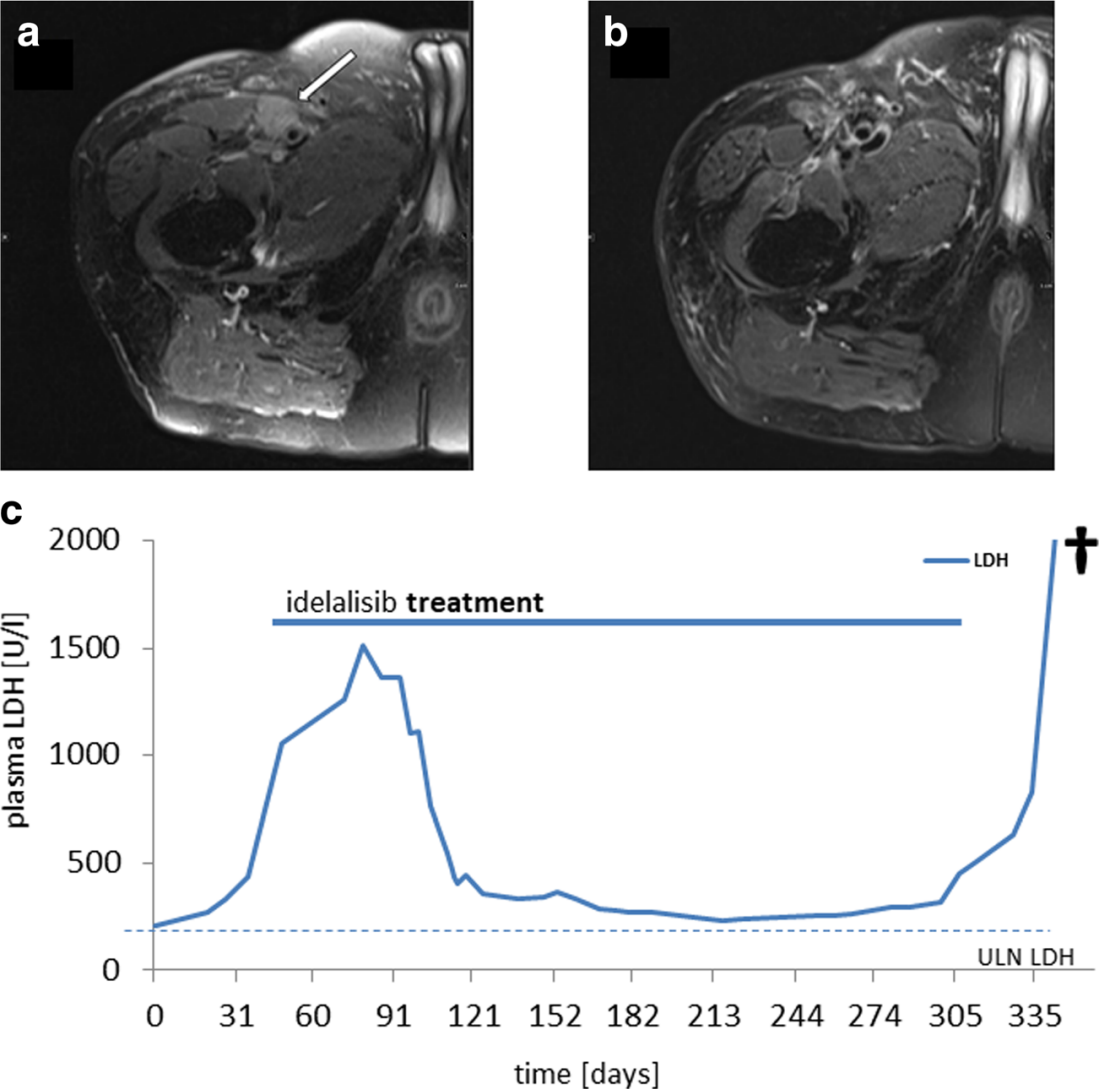
Pre-treatment MRI (**a**) demonstrates a well-defined slightly hyperintense soft tissue mass (arrow) between the musculus vastus medialis and the arteria femoralis superficialis on axial PD TSE FS transversal slices. At the 9-month MRI follow-up, (**b**) the tumor is no longer detectable, axial PD TSE FS transversal images showing post-therapeutic changes only. (**c**) Course of plasma LDH under idelalisib treatment plasma LDH levels as aggregated by routine laboratory monitored, local upper limit of normal (ULN) 256 [U/l] as indicated. Documentation starting (day 0) at 4 weeks after last prior treatment (radiotherapy), patient died on day 357 (†)

The initial diagnosis of stage IIIB BL disease and concurrent HIV-1 positivity was made in a 65-year-old male patient with multifocal lymphadenopathy and B-symptoms. The patient was started on immediate anti-viral therapy. At initial presentation, the patient appeared ineligible for multiphase leukemia-based regimens due to comorbidities (ischemic heart disease, impaired renal function), so that he received one course of R-CHOP, and upon adequate tolerance and response, further seven courses of R-CHOEP-21, inducing a complete remission. Ten months from initial diagnosis, relapse manifested as an isolated cutaneous manifestation (3 × 5 cm) in the upper thigh. Two courses of platine/Ara-C-based chemotherapy (R-DHAP) induced partial remission, consolidated by involved site radiotherapy. Fifteen months after initial diagnosis the disease recurred again, at this time refractory to methotrexate/Ara-C-based chemotherapy (dexamethasone 20 mg p.o., etoposide 60 mg/m^2^ d4 + 5 i.v., cytarabine 60 mg/m^2^ d4 + 5 i.v.; ifosfamide 400 mg/m^2^ d1–5 i.v.; methotrexate 500 mg/m^2^ i.v., methotrexate 12 mg i.th. d3). Additional radiotherapy and vinblastine every other week was started in palliative intention. Responding to the patient’s wish for further treatment, off-label idelalisib was initiated (150 mg bid) inducing shrinkage of the tumor (Fig. 1a, b) and decline of plasma LDH (Fig. 1c). Idelalisib was continued without infectious complications or adverse reactions for 10 months, until the tumor recurred and the patient succumbed to progressive disease.

Analysis of the initial lymphoma by high-throughput sequencing (HTS) detected a *TP53* mutation (*T175H*, VAF 83.5%), compatible with coincident structural loss of the second TP53 allele and presumably representing the major unfavorable molecular factor in this case [6–8]. In addition, we identified two mutations in the catalytic alpha subunit of the *PIK3CA* gene, i.e., p.H1047R (variant allele frequency (VAF) 5.0%) and p.Q1033R (VAF 3.7%) affecting exon 21 on the same allele. This *PIK3CA* H1047R mutation affects the highly conserved catalytic subunit p110a and has previously been identified at high frequency in many types of solid tumors [9, 10]. In patients receiving PI3K/AKT/mTOR inhibitors, the presence of the *PIK3CA* H1047R mutation was associated with improved response rates compared with patients with wild-type *PIK3CA* or other *PIK3CA* mutations (38% vs. 10% vs. 13%) [11]. So far, *PIK3CA* H1047R mutations have not been described in BL cell lines or primary tumor samples. However, preclinical evidence demonstrated that deregulated *MYC* and PI3K activity synergize in BL pathogenesis [8], and that pathway inhibitors suppress PI3K-dependent proliferation of human BL cells [12, 13]. We speculate, that in the case of the index patient, the *PIK3CA* H1047R mutation was a lymphoma propagating factor by cooperation of MYC/IgH and *PIK3CA* [14]*.*

In conclusion, our report supports further exploration of PI3K inhibition in the treatment of patients with refractory/relapsed BL. Screening for *PIK3CA* mutations may allow the detection of specific lymphoma cell vulnerabilities for targeted therapy in relapsed/refractory disease.
